# The 2′-*O*-methyladenosine nucleoside modification gene *OsTRM13* positively regulates salt stress tolerance in rice

**DOI:** 10.1093/jxb/erx061

**Published:** 2017-03-28

**Authors:** Youmei Wang, Dongqin Li, Junbao Gao, Xukai Li, Rui Zhang, Xiaohuan Jin, Zhen Hu, Bo Zheng, Staffan Persson, Peng Chen

**Affiliations:** 1 College of Plant Science and Technology, HuaZhong Agricultural University, Wuhan 430070, China; 2 Biomass and Bioenergy Research Centre, HuaZhong Agricultural University, Wuhan 430070, China; 3 College of Life Science, HuaZhong Agricultural University, Wuhan 430070, China; 4 College of Horticulture and Forestry Sciences, HuaZhong Agricultural University, Wuhan 430070, China; 5 School of Biosciences, University of Melbourne, Parkville 3010 VIC, Australia

**Keywords:** 2′-*O*-methyladenosine, ABA, modified nucleoside, rice, salt stress tolerance, tRNA.

## Abstract

Stress induces changes of modified nucleosides in tRNA, and these changes can influence codon–anticodon interaction and therefore the translation of target proteins. Certain nucleoside modification genes are associated with regulation of stress tolerance and immune response in plants. In this study, we found a dramatic increase of 2′-*O*-methyladenosine (Am) nucleoside in rice seedlings subjected to salt stress and abscisic acid (ABA) treatment. We identified *LOC_Os03g61750* (*OsTRM13*) as a rice candidate methyltransferase for the Am modification. *OsTRM13* transcript levels increased significantly upon salt stress and ABA treatment, and the OsTrm13 protein was found to be located primarily to the nucleus. More importantly, *OsTRM13* overexpression plants displayed improved salt stress tolerance, and vice versa, *OsTRM13* RNA interference (RNAi) plants showed reduced tolerance. Furthermore, *OsTRM13* complemented a yeast *trm13Δ* mutant, deficient in Am synthesis, and the purified OsTrm13 protein catalysed Am nucleoside formation on tRNA-Gly-GCC *in vitro*. Our results show that *OsTRM13*, encoding a rice tRNA nucleoside methyltransferase, is an important regulator of salt stress tolerance in rice.

## Highlight

*OsTRM13* is a 2′-*O*-methyladenosine tRNA nucleoside modification gene that positively regulates salt stress tolerance in rice.

## Introduction

Modified nucleosides are derivatives of the four common nucleosides, adenosine (A), guanosine (G), uridine (U), and cytidine (C). They are particularly important for transfer RNA (tRNA), since more than 85% of all modified nucleosides are present on tRNA molecules (RNA modification database, http://rna-mdb.cas.albany.edu/;Cantara *et al.*, 2011). Modified nucleosides influence the decoding process of tRNA, and therefore affect protein translation and cellular metabolism (Björk *et al.*, 1987; Bjork, 1995; Urbonavicius *et al.*, 2001). Approximately 25–30 different modified nucleosides can be found in tRNAs across organisms, and on average each tRNA molecule contains at least five to six modified nucleosides (Jühling *et al.*, 2009; Machnicka *et al.*, 2013). At present, more than 600 sequenced tRNAs are available from archea to eukaryotes, including cytosolic, mitochondrial and chloroplast tRNAs (Modomics, http://modomics.genesilico.pl/;Dunin-Horkawicz *et al.*, 2006). The modified nucleosides in tRNAs can change in response to alterations of environmental conditions, and across developmental stages, including aging, starvation, and different stress conditions (Dirheimer *et al.*, 1995; Suzuki and Nagao, 2011*b*; Dedon and Begley, 2014). Therefore, it has been suggested to work as a ‘sensing system’ to link environmental and developmental stimuli to cellular translational machinery and metabolism (Chan *et al.*, 2012; El Yacoubi *et al.*, 2012; Zinshteyn and Gilbert, 2013).

Recently, a mechanism in which tRNA-derived nucleoside modifications control the translation of stress-related proteins was proposed, and termed ‘MoTT’ (*m*odification *o*f *t*RNA *t*uned) (Chan *et al.*, 2012; Gu *et al.*, 2014). According to this model, cells respond to stresses (e.g. oxidative stress) by changes of tRNA nucleoside modifications, which subsequently influence decoding of certain codons and therefore the translation of proteins (Dedon and Begley, 2014). Nucleoside modification changes were therefore not only manifested as a ‘signal’ for stress, but may also be used as a ‘regulatory module’ to timely adapt the cell to environmental changes, in a fast, broad and effective manner (Chan *et al.*, 2010, 2012). Such a regulation also plays an important role in the development of some human diseases (Dirheimer *et al.*, 1995; Kirino *et al.*, 2004; Rodriguez *et al.*, 2007; Suzuki and Nagao, 2011*a*).

Knowledge of tRNA nucleoside modification genes in higher plants is rather limited. Among 642 sequenced tRNAs in the Modomics database, only 78 are from land plants. According to the PlantRNA database (http://plantrna.ibmp.cnrs.fr/;Cognat *et al.*, 2013), more than 600 tRNA genes are present in the Arabidopsis genome coding for *ca* 200 tRNA unique sequences, of which none is associated with information of modified nucleosides. Nevertheless, several mutants of tRNA nucleoside modification genes have been described in Arabidopsis, many of which result in retarded plant growth (Chen *et al.*, 2006; Hu *et al.*, 2010; Zhou *et al.*, 2013), impaired immune response (Wang *et al.*, 2013) or abrogated abiotic stress tolerance (Zhou *et al.*, 2013; Burgess *et al.*, 2015).

Rice is an important cereal crop as well as a monocot model plant, and stress tolerance is a very important trait for breeding purposes. Rice is one of the most salt sensitive cereal crops, contrasting with, for example, barley, which is one of the most salt tolerant (Munns and Tester, 2008). Different plant species develop specific strategies to combat abiotic stress, and insights into these strategies have been obtained via metabolite comparisons between stress-tolerant and stress-sensitive accessions (Hasegawa *et al.*, 2000; Zhu, 2002). Plants accumulate several osmolytes in response to drought and salt stress, including soluble sugars (glucose, sucrose, trehalose), non-digestible carbohydrates (e.g. raffinose, stachyose, and verbascose), polyols (e.g. mannitol and sorbitol), amino acids (e.g. proline), quaternary ammonium compounds (e.g. glycine betaine) and polyamines (e.g. putrescine, spermidine and spermine) (Golldack *et al.*, 2014; Jorge *et al.*, 2016). These compounds, in one way or another, help the cell to maintain turgor pressure and avoid water loss, and also to deal with ROS and re-establish redox balance (Munns and Tester, 2008). The response of amino acids, soluble sugars and TCA cycle intermediates from different rice cultivars to salt does in general coincide with the core metabolite adaptation in most glycophylic plants, such as *Arabidopsis thaliana* (Jorge *et al.*, 2016). Abscisic acid (ABA) plays an important role in abiotic stress signaling (Zhu, 2002). The connection between ABA and abiotic stress, mostly drought and salt stress, is well established in Arabidopsis but less well in rice. Many genes and proteins are involved in the ABA-mediated stress-signaling pathway, including ABA biosynthesis (*ABA1*, *AAO3*, *ABA3*, *NCED*s), ABA receptor (*PYR*/*PYL*/*RCAR*) and PP2C (e.g. *SnRK2*), AP2 (e.g. *DREB2A*, *ABI4*) and bZIP transcription factor (*ABF*s, *ABI5*) genes (Ye *et al.*, 2012; Nakashima and Yamaguchi-Shinozaki, 2013).

Although several studies have shown that tRNA nucleoside modification genes can influence plant growth, anthocyanin biosynthesis, hormone homeostasis and immune response in Arabidopsis (Chen *et al.*, 2006; Zhou *et al.*, 2009; Hu *et al.*, 2010; Nelissen *et al.*, 2010; Leihne *et al.*, 2011; Xu *et al.*, 2012), similar studies are lacking for crop plants, including rice. Indeed, no sequenced tRNA was available for rice in the PlantRNA, Modomics or RNAmod databases, nor for tRNA nucleoside modification genes. In this study we found a significant increase of 2′-*O*-methyladenosine (Am) nucleosides during salt stress and ABA treatment in rice. We further identified LOC_Os3g61750 (*OsTRM13*) as the gene responsible for Am modification. In accordance with the elevated Am nucleoside levels, *OsTRM13* transcript levels increased upon salt stress and ABA treatment. In addition, transgenic rice overexpressing *OsTRM13* showed improved salt tolerance. These data indicate that *OsTRM13* is responsible for tRNA nucleoside modification, and that this function is important for salt stress and ABA hormone responses in rice.

## Materials and methods

### Plant material and bacterial strains

Nipponbare rice (*O. sativa* L. spp*. japonica*) was used throughout this study. Minghui63 (MH63) and Zhanshan97 (ZS97) rice were kindly provided by Dr Liangcai Peng from Huazhong Agricultural University, and *Brachypodium distachyon* (L.) was provided by Dr Lingqiang Wang in Huazhong Agricultural University. Yeast strains Y07126 and Y27126 were purchased from EUROSCCARF (www.euroscarf.de). *Arabidopsis thaliana* Columbia ecotype, and hybrid poplar 717 (*Populus tremula* × *P. alba* 717-1B4 genotype) were maintained in our own lab.

### Sampling of rice tissues upon abiotic stress or ABA hormone treatment

Seeds of Nipponbare rice (NPB) were sterilized with 70% ethanol followed by 2.5% sodium hydrochloride, washed and soaked in distilled water for 2 d. Seeds were grown hydroponically in a climate chamber with 16 h–8 h light–dark photoperiod at 28 °C. Ten-day-old rice seedlings were used as starting material for salt stress and ABA treatment. Cold stress was applied by transferring seedlings into pre-equilibrated 4 °C cold distilled water in a cold room for up to 7 d, drought/air-drying stress was performed by transferring seedlings onto Whatman 3MM filter paper (GE Healthcare Life Sciences) in a growth chamber for up to 7 d. Salt stress was applied by changing distilled water to 200 mM NaCl solution for rice seedling cultivation. ABA treatment was performed by applying 100 μM ABA in culture medium. Samples were taken at specified time points in triplicates, flash-frozen in liquid nitrogen and stored at –80 °C until further use.

### tRNA isolation and nucleoside analysis by liquid chromatography–mass spectrometry

Small RNAs were extracted using microRNA Extraction Kit (Omega Bio-tek Inc.). RNA concentration was determined using a NanoDrop ND-1000 spectrophotometer (Thermo Scientific). About 20 µg tRNA was digested with 2 units of P1 nuclease (Sigma-Aldrich) and 1.5 unit of calf intestine alkaline phosphatase (Toyobo) in 20 mM Hepes–KOH (pH 7.0) at 37 °C for 3 h (Noma *et al.*, 2006). Samples were diluted with Milli-Q water (Millipore Synergy) to a concentration of 10 µg ml^–1^.

Detailed settings for each nucleoside are summarized in Supplementary Table S1 at *JXB* online. An API 4000 Q-Trap mass spectrometer (Applied Biosystems) was used with an LC-20A HPLC system and a diode array UV detector (190−400 nm). Electrospray ionization mass spectrometry was conducted in positive ion mode. The nebulizer gas, auxiliary gas, curtain gas, turbo gas temperature, entrance potential, and ion spray voltage were 60 psi, 65 psi, 15 psi, 550 °C, 10 and 5500 V, respectively. An Inertsil ODS-3 column (2.1 mm×150 mm, 5 µm particle size; Shimadzu) was used for nucleoside separation. The mobile phase gradient was the following (Yan *et al.*, 2013): 0−10 min, 0−50% solvent B; 10−13 min, 50−100% solvent B; 13−23 min, 100% solvent B; 23−23.1 min, 100−5% solvent B; 23.1−30 min, 5–0% solvent B. The ﬂow rate was 0.6 ml min^–1^ at ambient temperature. The injection volume was 10 µl. Multiple reaction monitoring mode was performed to determine parent-to-product ion transitions. Uridine, cytosine, adenosine, guanosine, 7-methylguanosine, 5-methyluridine, 5-methylcytidine, and 2′-*O*-methylguanosine nucleoside standards were purchased from Santa Cruz Biotechnology (Dallas, TX, USA).

### Protein purification and tRNA *in vitro* methylation

*OsTRM13* full length cDNA was amplified and cloned into pGEX-6P-3 (GE healthcare Life Sciences, Shanghai, China) using *Bam*HI and *Not*I sites, resulting in a fusion protein with glutathione *S*-transferase (GST) at the N-terminus. The recombinant vector was transformed into BL21 cells; expression of fusion protein was induced with 0.5 µM isopropyl β-D-1-thiogalactopyranoside (IPTG) and purified with ProteinIso GST Resin (Transgen Biotech). The N-terminal GST tag was cleaved off by ProScission Protease (Genscript Biotechnology Co. Ltd, Nanjing, China). Tag-free protein was eluted in the presence of 50 mM Tris–HCl (pH 7.0), 150 mM NaCl, 1 mM EDTA, and 1 mM DTT.

Yeast tRNA-Gly-GCC was synthesized in pGEM-T easy vector (Promega, Beijing, China). tRNA-Gly-GCC was *in vitro* transcribed with Riboprobe *in vitro* Transcription Systems (Promega). Buffers for tRNA methylation were described previously (Wilkinson *et al.*, 2007), with 0.5 mM AdoMet as methyl donor. The substrate tRNA was provided in a final concentration of 1 or 2 µM (designated as + or ++), and the final concentration of OsTRM13 protein was 2 or 10 µM (designated as + or ++).

### Yeast complementation

*OsTRM13* full length cDNA was amplified and cloned with *Sma*I and *Kpn*I restriction sites in pAUR101, a chromosomal integrating vector for *Saccharomyces cerevisiae* (Takara). The resulting plasmid was transformed into *Δtrm13* mutant strain (Y07126: *MATa; ura3Δ0; leu2Δ0; his3Δ1; met15Δ0; YOL125w (TRM13)::kanMX4*), and congenic *trm13*^*+*^ wild-type strain Y27126 (*MATa/MATα; ura3Δ0/ura3Δ0; leu2Δ0/leu2Δ0; his3Δ1/his3Δ1; met15Δ0/MET15; LYS2/lys2Δ0; YOL125w/YOL125w (TRM13)::kanMX4*) served as control. The recombinant vector was transformed into Y07126 and selected on YPD medium with 1.5 µg ml^–1^ Aureobasidin A (Yeasen Biotech Co. Ltd, Shanghai, China). Yeast strains carrying the recombinant plasmid (Y07126+OsTRM13), Y07126 and Y27126 were analysed further for growth phenotype on YPD medium, and Am nucleoside level by liquid chromatography–mass spectrometry (LC-MS).

### Subcellular localization

OsTRM13-eGFP vector was constructed for subcellular localization. The enhanced green fluorescent protein (eGFP) tag was fused in frame to the 3′-end of the *OsTRM13* gene sequence. Primers are listed in Supplementary Table S2. The construct was transformed into *Agrobacterium* strain GV3101 and infiltrated into tobacco leaves for confocal microscopy. The subcellular localization of GFP was visualized using a confocal laser scanning microscope (Leica SP5 CLSM) with ×63 objective lens. 4′,6-Diamidino-2-phenylindole (DAPI) staining was used as a nuclear marker.

### Vector construction for overexpression and RNAi transgenic rice

The full-length *OsTRM13* cDNA sequences were amplified and cloned with *Kpn*I and *Xba*I sites into a pD1301s-eGFP binary vector. This vector also carried hygromycin as a plant selection marker, and the eGFP gene between *Sal*I and *Pst*I restriction sites. The recombinant pD1301s-OsTRM13-eGFP was used both for overexpression and subcellular localization. For RNAi construction, a 162 bp fragment was amplified and cloned into gateway RNAi destination vector pH7GWIWG2(II) (http://gateway.psb.ugent.be/) via pENTR/D-TOPO entry vector (Thermo Fisher Scientific, China) (Karimi *et al.*, 2007).

Overexpression and RNAi destination vector was introduced into *Agrobacterium tumefaciens* strain EHA105 and transformed into Nipponbare rice. Positive T0 transgenic lines were selected using hygromycin (Hyg) as plant selection marker, and segregating T1 plants were screened by PCR and verified by qRT-PCR.

### Chlorophyll and proline measurement in transgenic rice before and after salt stress

Flag leaves at bolting stage were cut into pieces and submerged in distilled water or 200 mM NaCl solution for 3 d. Leaf sample was ground and extracted for chlorophyll content determination (Inskeep and Bloom, 1985; Srivastava *et al.*, 2016). For proline content determination before and after salt stress, 2-week-old transgenic or NPB seedlings cultivated in a greenhouse were subjected to 200 mM NaCl for 3 d, and proline content was measured according to Bates (1973).

### Enzymatic activity of superoxide dismutase and peroxidase in transgenic rice before and after salt stress

Two-week-old pD1301s-OsTRM13-eGFP(overexpression) or pH7GWIWG2(II)-OsTRM13(RNAi) transgenic seedlings cultivated in a greenhouse were subjected to salt stress in 200 mM NaCl for 5 d, samples were taken at 0, 3, and 5 d, and three biological replicates were used from each line. Peroxidase (POD) assay kit (Nanjing Jiancheng Bioengineering Institute, China) was used for POD enzymatic activity determination, following the method of Amako *et al.* (1994). A superoxide dismutase (SOD) assay kit (Nanjing Jiangcheng Bioengineering Institute) was used based on the hydroxylamine method (Zhang, 2008). One unit of SOD activity is defined as the amount of SOD enzyme required for 50% inhibition of superoxide anion radicals determined by colorimetric method in a 1 ml reaction (Zhang *et al.*, 2008). The POD or SOD activities were presented as U mg^–1^ proteins.

### Quantitative RT-PCR

Total RNA was extracted by the RNAprep pure Plant Kit (Tiangen Biotech, Beijing, China). M-MLV RTase (TaKaRa, Dalian, China) was used to generate cDNA. qRT-PCR was conducted using a Bio-Rad IQ5^TM^ real-time PCR system (Life Science, Wuhan, China). *LOC_Os06g48970* (*UBQ*) and *LOC_Os06g11170* (*ACTIN*) were served as reference genes (Narsai *et al.*, 2010). The ΔΔ*C*_t_ method was used for quantification of relative expression (Livak and Schmittgen, 2001).

At least three biological replicates were used for each line analysed, and mean and standard deviation (SD) were calculated. Student’s *t*-test was performed to judge the difference significance level (*, statistically significant at *P*≤0.05; **, statistically significant at *P*≤0.01).

## Results

### Salt stress and ABA treatment induced a significant increase in 2′-*O*-methyladenosine nucleosides in rice

**Fig. 1.**
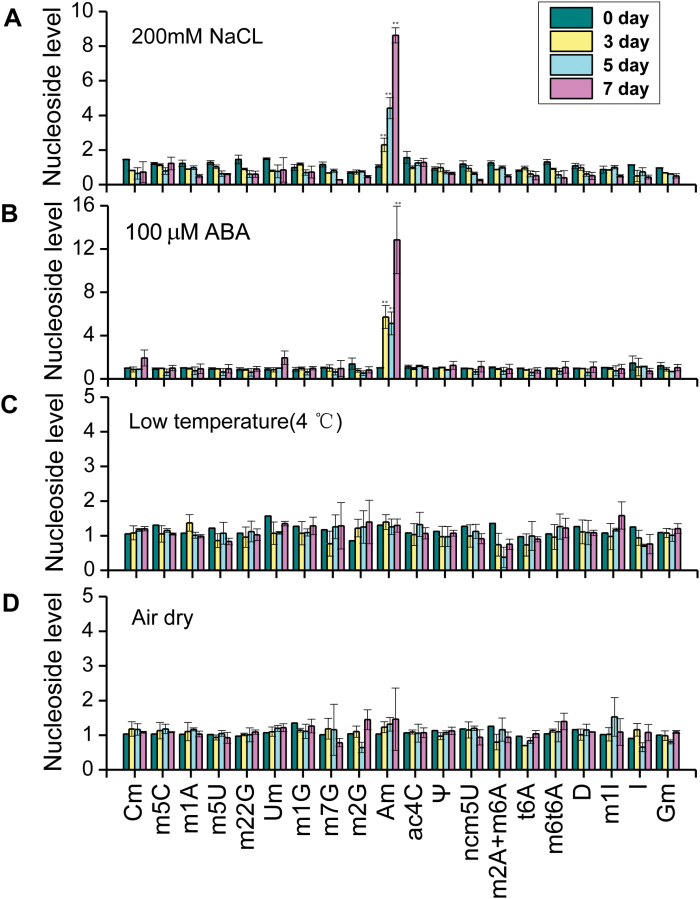
tRNA nucleoside modifications are induced during stress and ABA treatment in rice. (A) Salt stress; (B) ABA treatment; (C) cold stress; (D) drought stress. Ten-day-old rice seedlings were used for various treatments. At each time point three biological replicates were collected, each replicate contained 20 seedlings. Total tRNA was prepared and digested into nucleosides and analysed by LC-MS. The fold change for each nucleoside under stress condition compared with normal condition is presented. Modified nucleosides are shown in abbreviated form. m^2^A and m^6^A were not separated well in LC-MS and therefore the sum of these two peaks is presented. Error bars represent standard deviation from three biological replicates. *P<0.05 and **P<0.01 by Student’s *t*-test.

Modified nucleosides in tRNAs may change dramatically during various stress conditions in animal and yeast systems (Chan *et al.*, 2012; Dedon and Begley, 2014); however, similar studies in plants are lacking. To assess modified nucleosides in tRNAs, 9-day-old seedlings of Nipponbare rice (NPB) were subjected to drought (air dry), cold, and salt stress or abscisic acid (ABA) hormone treatment, and were analysed by LC-MS for modified nucleosides (see Supplementary Table S1). Nucleoside levels were calculated by their abundance in stress conditions divided by that in control condition (Fig. 1). A prominent increase of 2′-*O*-methyladenosine (Am) nucleosides was observed during salt stress and ABA treatment (Fig. 1A, B). Am nucleoside level increased from 3 to 7 d during salt stress, and its abundance peaked at 7 d where it was 10 times higher than that of control (Fig. 1A). The Am nucleoside profile showed a similar trend for ABA-treated samples, which also showed highest abundance after 7 d treatment (Fig. 1B). In contrast, Am nucleoside levels did not change significantly during cold or drought stress conditions (Fig. 1C, D).

### Identification of a putative 2′-*O*-methyladenosine modification gene in rice

Am nucleoside may be found at position 4 in eukaryotic tRNAs (Wilkinson *et al.*, 2007). Trm13p of *Saccharomyces cerevisiea* (baker’s yeast) was the first enzyme identified for Am4 and Cm4 modifications (Wilkinson *et al.*, 2007). Trm13p belongs to the Rossman fold (RFM) group of *S*-adenosyl-methionine (AdoMet)-dependent methyltransferases (MTases) (Schubert *et al.*, 2003). Using AdoMet as a methyl donor, Trm13p catalyses Am or Cm formation at position 4 on tRNA-His, tRNA-Pro and tRNA-Gly in yeast (Wilkinson *et al.*, 2007). Trm13 proteins are unique for eukaryotes, and amino acid residues critical for AdoMet binding and conformation of the catalytic domain have been suggested (Tkaczuk, 2010).

**Fig. 2.**
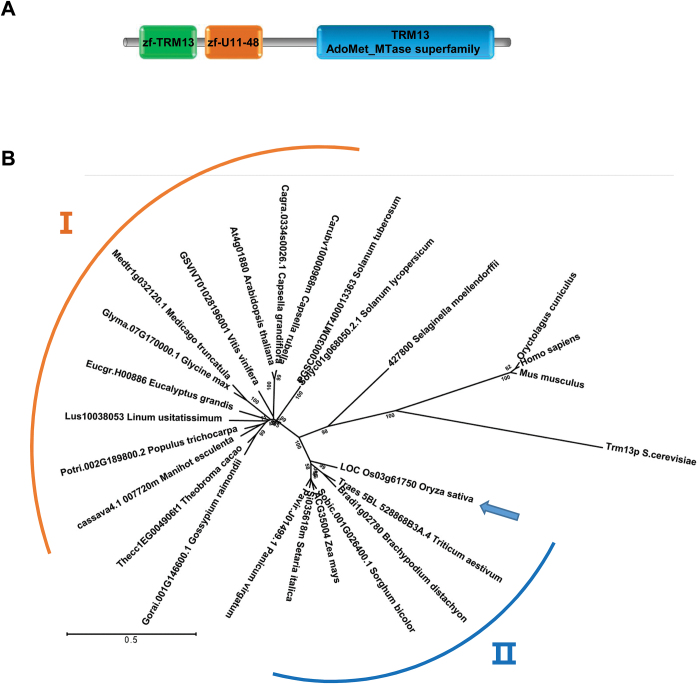
Domain structure and phylogenetic tree of OsTrm13 and related proteins. (A) OsTrm13 protein domain prediction from Pfam database (http://pfam.xfam.org/). zf-TRM13 and zf-U11-48 are zinc finger domains, TRM13 is an AdoMet MTase domain. (B) Neighbor joining tree of Trm13p homologs. Numbers at each branching point are supporting values from bootstrap analysis (1000 iterations). Both protein accession numbers and organism names are shown. I and II represent two clusters of Trm13p homologs in land plants. (This figure is available in color at *JXB* online.)

No components for Am and/or Cm nucleoside modification have been reported for plants. Therefore, we used the yeast Trm13p protein sequence to find Am modification candidate genes in the rice genome. LOC_Os03g61750 was the only candidate with a blastp value below 1.0 × 10^–6^ (data not shown), and we therefore tentatively named this gene *OsTRM13*. Indeed, OsTrm13 was annotated as methyltransferase-Trm13-domain-containing protein in the RGAP database (http://rice.plantbiology.msu.edu/). In addition to the TRM13 MTase domain, OsTrm13 also contained two zinc-finger domains, zf-Trm13-CCCH and zf-U11-48K (Fig. 2A). The OsTrm13 showed 28.9% sequence similarity to Trm13p, and 54.5% to a putative Arabidopsis homolog (At4g01880). In a phylogenetic tree of eukaryotic Trm13p homologs, the mammal and yeast Trm13s were sparsely separated from the plant Trm13 homologs. Plant Trm13p homologs were divided into two clades, where the Arabidopsis Trm13p homolog At4g01880 was in group I and OsTrm13 was in group II (Fig. 2B).

### OsTrm13 protein is located in the nucleus

**Fig. 3.**
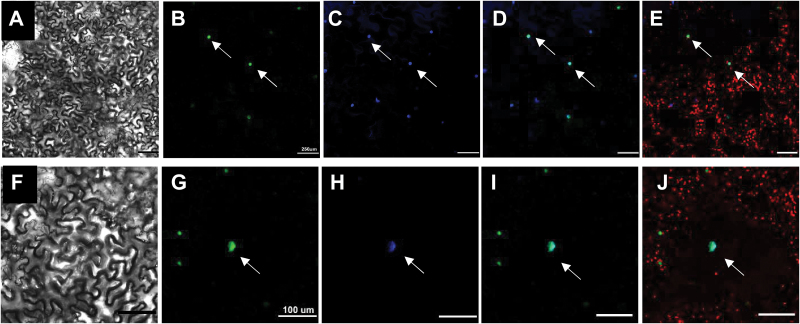
Subcellular localization of OsTrm13-eGFP fusion protein by tobacco leaf transient transformation. (A, F), bright field; (B, G), eGFP channel; (C, H), DAPI staining; (D, I), merge of eGFP and DAPI channels; (E, J), chlorophyll autofluorecence. (A–E), scale bar: 250 μm; (F–J), scale bar: 100 μm.

An eGFP tag was fused to the C-terminus of OsTrm13 protein to investigate the subcellular localization of the protein in transient transfected tobacco leave cells (Fig. 3). As eGFP fluorescence signal was observed in a compartment reminiscent of the nucleus (Fig. 3B, G), DAPI staining was used to verify the nuclear localization of OsTrm13-eGFP (Fig. 3C, H). Although OsTrm13 was predicted to be localized to the chloroplast by TargetP (http://www.cbs.dtu.dk/services/TargetP/), we did not find any overlap between the eGFP signal and chlorophyll autofluorecence (Fig. 3E, J). Our data are, furthermore, in agreement with the function of Trm13 in yeast and animal cells where it acts as tRNA nucleoside modifier in the cytosol and nucleus (Wilkinson *et al.*, 2007; Tkaczuk, 2010).

### OsTrm13 catalyses formation of 2′-*O*-methylated nucleosides *in vitro*

Am nucleoside is only found in tRNA-His-GUG in *S. cerevisiea*; however, no tRNA-His-GUG from Arabidopsis or rice contains an adenosine at position 4 (Table 1). Since tRNA-Pro-UGG and tRNA-Gly-GCC also are Trm13p substrates (Wilkinson *et al.*, 2007), yeast tRNA-Gly-GCC was used as substrate for *in vitro* methylation assays.

**Table 1.** DNA sequences of tRNA-His-GTG, tRNA-Gly-GCC and tRNA-Pro-TGG in yeast, Arabidopsis and rice AA, amino acid; AC, anticodon; Acc, acceptor; Ac, anticodon; V, variable loop; T, TψC loop; Sc, *S. cerevisiae*; At, *A. thaliana*, Os, *O. sativa*.

Species	Chr	AA	AC	p-1	Acc	p8	D	D	D	p26	Ac	Ac	Ac	V Region	T	T	T	Acc	p73	CCA
					Stem	p9	Stem	Loop	Stem		Stem	Loop	Stem		Stem	Loop	Stem	Stem		
Sc		His	GTG		GCC**A**TCT	TA	GTAT	AGTGGTTA	GTAC	A	CATCG	TT**GTG**GC	CGATG	AAAC	CCTGG	TTCGATT	CTAGG	AGATGGC	A	CCA
At	nucleus	His	GTG	G	GTG**G**CTG	TA	GTTT	AGTGGTAA	GAAT	T	CCACG	TT**GTG**GC	CGTGG	AGAC	CTGGG	CTCGAAT	CCCAG	CAGCCAC	A	CCA
At	mitochondrion	His	GTG	G	GCG**G**ATG	TA	GCC	AAGTGGATCAA	GGC	A	GTGGA	TTG**TG**AA	TCCAC	CATGC	GCGGG	TTCAATT	CCCGT	CGTTCGC	C	CCA
At	nucleus	His	GTG	G	GTG**G**CTG	TA	GTTT	AGTGGTTA	GAAT	T	CCACG	TT**GTG**GC	CGTGG	AGAC	CTGGG	CTCGAAT	CCCAG	CAGCCAC	A	CCA
At	nucleus	His	GTG	G	GTG**G**TTG	TA	GTAT	AGCGGTTA	GTAT	C	CCACG	TT**GTG**GC	CGTGG	GGAC	CCGGG	CTCGAAT	CCCGG	CAGCCAC	A	CCA
At	nucleus	His	GTG	G	GTG**G**CTG	TA	GTTT	AGTGGTTA	GAAT	C	CCACG	TT**GTG**GC	CGTGG	GGAC	CTGGG	CTCGAAT	CCCAG	CAGCCAC	A	CCA
At	chloroplast	His	GTG	G	GCG**G**ATG	TA	GCC	AAGTGGATTAA	GGC	A	GTGGA	TT**GTG**AA	TTCAC	CATC	GCGGG	TTCAATT	CCCGT	CGTTCGC	C	CCA
At	chloroplast	His	GTG	G	GCG**G**ATG	TA	GCC	AAGTGGATCAA	GGC	A	GTGGA	TT**GTG**AA	TCCAC	CATGC	GCGGG	TTCAATT	CCCGT	CGTTCGC	C	CCA
Os	mitochondrion	His	GTG	G	GCG**G**ATG	TA	GCC	AAGTGGATCAA	GGC	A	GTGGA	TT**GTG**AA	TCCAC	CATGC	GCGGG	TTCAATT	CCCGT	CGTTCGC	C	CCA
Os	nucleus	His	GTG	G	GTG**G**CTG	TA	GTTT	AGTGGTGA	GAAT	T	CCACG	TT**GTG**GC	CGTGG	AGAC	CTGGG	CTCGAAT	CCCAG	CAGCCAC	A	CCA
Os	nucleus	His	GTG	G	GTG**G**CTG	TA	GTTT	AGTGGTGA	GAAT	T	CTACG	TT**GTG**GC	CGTAG	AGAC	CTGGG	CTCGAAT	CCCAG	CAGCCAC	A	CCA
Sc		Gly	GCC		GCG**C**AAG	TG	GTTT	AGTGGTA	AAAT	C	CAACG	TT**GCC**AT	CGTTG	GGCC	CCCGG	TTCGATT	CCGGG	CTTGCGC	A	CCA
At	nucleus	Gly	GCC		GCA**C**CAG	TG	GTCT	AGTGGTA	GAAT	A	GTACC	CT**GCC**AC	GGTAC	AGAC	CCGGG	TTCGATT	CCCGG	CTGGTGC	A	CCA
At	nucleus	Gly	GCC		GCA**C**CAG	TG	GTC	TAGTGGCAT	GAT	A	GTACC	CT**GCC**AC	GGTAC	AGAC	CCGGG	TTCAATT	CCCGG	CTGGTGC	A	CCA
At	nucleus	Gly	GCC		GCA**C**CAG	TG	GTC	TAGTGGCAT	GAT	A	GTACC	CT**GCC**AC	GGTAC	ATAC	CCGGG	TTCAATT	CCCGG	CTGGTGC	A	CCA
At	nucleus	Gly	GCC		GCA**C**CAG	TG	GTCT	AGTGGTA	GAAT	A	GTACC	CT**GCC**AC	GGTAC	AGAC	CCGGG	TTCAATT	CCCGG	CTGGTGC	A	CCA
At	nucleus	Gly	GCC		TAA**C**CAG	TG	GTCT	AGTGGTA	GAAT	A	GTACC	CT**GCC**AC	GGTAC	AGAC	CCGGG	TTCGATT	CCCGG	CTGGTGC	A	CCA
At	nucleus	Gly	GCC		GCA**C**CAG	TG	GTCT	AGTGGTA	GAAT	A	GTACT	CT**GCC**AC	GGTAC	AGAC	CCGGG	TTCGATT	CCCGG	CTGGTGC	A	CCA
At	mitochondrion	Gly	GCC		GCG**G**AAA	TA	GCTT	AATGGTA	GAGC	A	TAGCC	TT**GCC**AA	GGCTA	AGGTT	GAGGG	TTCAAGT	CCCTC	CTTCCGC	T	CCA
At	chloroplast	Gly	GCC		GCG**G**ATA	TA	GT	CGAATGGTAAA	AT	T	TCTCC	TT**GCC**AA	GGAGA	AGAC	GCGGG	TTCGATT	CCCGC	TATCCGC	C	CCA
Os	nucleus	Gly	GCC		GCA**C**CAG	TG	GTCT	AGTGGTA	GAAT	A	GTACC	CT**GCC**AC	GGTAC	AGAC	CCGGG	TTCGATT	CCCGG	CTGGTGC	A	CCA
Os	chloroplast	Gly	GCC		GCG**A**GCG	TA	GTT	CAATGGTAA	AAC	A	TCTCC	TT**GCC**AA	GGAGA	AGAT	ACGGG	TTCGATT	CCCGC	CGCTCGC	C	CCA
Os	nucleus	Gly	GCC		GCG**C**CAG	TG	GTCT	AGTGGTA	GAAT	A	GTACC	CT**GCC**AC	GGTAC	AGAC	CCTGG	TTCGATT	CCTTG	CTGGTGC	A	CCA
Os	nucleus	Gly	GCC		GCA**C**CAG	TG	GTCT	AGTGGTA	GAAT	A	GTACC	CT**GCC**AC	GGTAC	AGAC	CCGGG	TTCGATT	CCAGG	CTGGTGC	A	CCA
Os	nucleus	Gly	GCC		GCA**C**CAG	TG	GTCT	AGTGGTA	GAAT	A	GTACC	CT**GCC**AC	GGTAC	AGAC	CCGGG	TTCGTTT	CCCGG	CTGGTGC	A	CCA
Os	nucleus	Gly	GCC		GCA**C**CAG	TG	GTCT	AGTGGTA	GAAT	A	GTACC	CT**GCC**AC	GGTAC	AGAC	CCGTG	TTCGATT	CCCGG	CTGGTGC	A	CCA
Os	nucleus	Gly	GCC		GCA**C**CAG	TG	GTCT	AGTGGTA	GAAT	A	GTACC	CT**GCC**AC	GGTAC	GGAC	CCGG	TTTCGATTC	CCGG	CTGGTGC	A	CCA
Sc		Pro	TGG		GGG**C**GTG	TG	GTCT	AGTGGTA	TGAT	T	CTCGC	TT**TGG**GT	GCGAG	AGGCC	CTGGG	TTCAATT	CCCAG	CTCGCCC	C	CCA
At	nucleus	Pro	TGG		GGG**C**ATT	TG	GTC	TAGTGGTAT	GAT	T	CTCGC	TT**TGG**GT	GCGAG	AGGTC	CCGAG	TTCGATT	CTCGG	AATGCCC	C	CCA
At	chloroplast	Pro	TGG		AGG**G**ATG	TA	GCGC	AGCTTGGTA	GCGC	G	TTTGT	TT**TGG**GT	ACAAA	ATGTC	ACGGG	TTCAAAT	CCTGT	CATCCCT	A	CCA
At	mitochondrion	Pro	TGG		CGA**G**GTG	TA	GCGC	AGTCTGGTCA	GCGC	A	TCTGT	TT**TGG**GT	ACAGA	GGGCC	ATAGG	TTCGAAT	CCTGT	CACCTTG	A	CCA
At	nucleus	Pro	TGG		CAG**C**ATT	TG	GTC	TAGTGGTAT	GAT	T	CTCGC	TT**TGG**GT	GCAAG	AGGTC	CCGAG	TTCGATT	CTCGG	AATGCCC	C	CCA
At	nucleus	Pro	TGG		GGG**C**ATT	TG	GTC	TAGTGGTAT	GAT	T	CTCGC	TT**TGG**GT	GCGAG	AGGTC	CCGAG	TTCGATT	CTCGC	AATGCCC	C	CCA
Os	nucleus	Pro	TGG		GGG**C**ATT	TG	GTC	TAGTGGTAT	GAT	T	CTCGC	TT**TGG**GT	GCGAG	AGGTC	CCGAG	TTCGATT	CTCGG	AATGCCC	C	CCA
Os	mitochondrion	Pro	TGG		CGA**G**GTG	TA	GCGC	AGTCTGGTCA	GCGC	A	TCTGT	TT**TGG**GT	ACAGA	GGGCC	ATAGG	TTCGAAT	CCTGT	CACCTTG	A	CCA
Os	nucleus	Pro	TGG		GGG**C**GTT	TG	GTC	TAGTGGTAT	GAT	T	CTCGC	TT**TGG**GT	GCGAG	AGGTC	GCGAG	TTCGATT	CTCGC	AACGCCC	C	CCA
Os	chloroplast	Pro	TGG		AGG**G**ATG	TA	GCGC	AGCTTGGTA	GCGC	G	TTTGT	TT**TGG**GT	ACAAA	ATGTC	ACAGG	TTCAAAT	CCTGT	CATCCCT	A	CCA
Os	nucleus	Pro	TGG		GGGCATT	TG	GTC	TAGTGGTAT	GAT	T	CTCGC	TT**TGG**GT	GCGAG	AGGTC	CCGAG	TTCAATT	CTCGG	AATGCCC	C	CCA
Os	mitochondrion	Pro	TGG		AGG**G**ATG	TA	GCGC	AGCTTGGTA	GCGC	G	TTCGT	TT**TGG**GT	ACAAA	ATGTC	ACGGG	TTCAAAT	CCTGT	CATCCCT	A	CCA
Os	mitochondrion	Pro	TGG		AGG**G**ATG	TA	GCGC	AGCTTGGTA	GCGC	G	TTCGT	TT**TGG**GT	ACAAA	ATGTC	ACGGG	TTCAAAT	CCTGT	CATCCCT	A	CCA

**Fig. 4.**
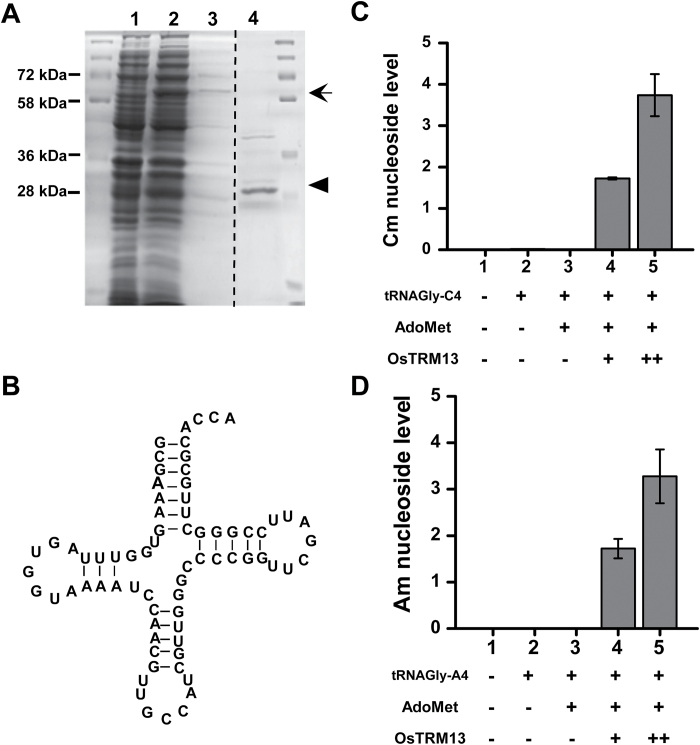
OsTrm13 can methylate tRNA-Gly-GCC *in vitro*. (A) *In vitro* expression of GST-tagged OsTrm13 and purification of tag-free OsTrm13 protein. Protein sizes are shown on the left. Lane 1: total protein from cell extract. Lane 2: total protein after IPTG induction. Position of GST-OsTrm13 is indicated with arrow. Lane 3: purified GST-OsTrm13 protein from the GST column. Lane 4: purified tag-free OsTrm13 protein after ProScission Protease digestion. The expected size of GST-OsTrm13 is indicated with an arrow, and tag-free OsTrm13 protein with an arrowhead. Dotted lines indicate separate gels. (B) Cloverleaf structure of substrate tRNA: yeast tRNA-Gly-GCC. Adenosine at position 4 is highlighted. (C) Cm nucleoside level during *in vitro* methylation with tRNA-Gly-C4 (cytidine at position 4) as substrate. (D) Am nucleoside level during *in vitro* methylation with tRNA-Gly-A4 (adenosine at position 4) as substrate. Error bars represent standard deviation from two technical replicates.

OsTrm13 was expressed as an N-terminal fusion protein with GST. Expression of this fusion protein (~66 kDa) was induced by IPTG and tag-free protein (~30 kDa) was purified after ProScission Protease cleavage (Fig. 4A). Yeast tRNA-Gly-GCC was *in vitro* transcribed as a naked/unmodified transcript from linearized pGEM-T vector (Fig. 4B). Purified OsTrm13 protein was added to the methylation reaction, with AdoMet as methyl donor (Wilkinson *et al.*, 2007). The products were digested into nucleosides and analysed by LC-MS. As shown in Fig. 4C, Cm production was positively correlated with OsTrm13 protein amount and input of tRNA. When the cytidine at position 4 was mutated to adenosine, OsTrm13-dependent Am nucleoside was formed instead (Fig. 4D). In the absence of OsTrm13 protein, neither Cm nor Am was found, and the reaction needed AdoMet as methyl donor (Fig. 4C, D). These results corroborated that OsTrm13 is an AdoMet-dependent MTase that could methylate A to Am or C to Cm nucleoside on yeast tRNAs *in vitro*.

### Complementation of yeast *Δtrm13* mutant by *OsTRM13*

**Fig. 5.**
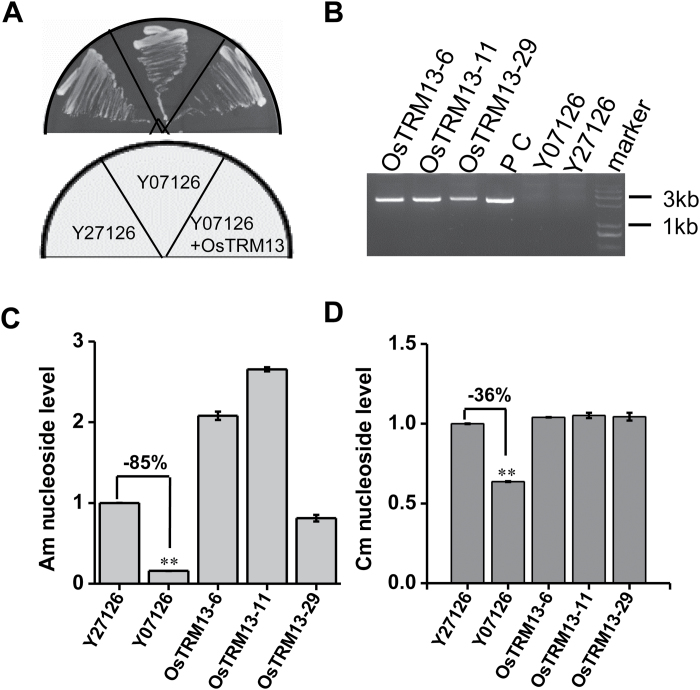
OsTrm13 can complement a yeast *trm13* mutant. (A) Growth phenotype comparison of the different yeast strains. Y07126 (*trm13* mutant): *MATa; ura3Δ0; leu2Δ0; his3Δ1; met15Δ0; YOL125w (TRM13)::kanMX4*; Y27126 (wild type): *MATa/MATα; ura3Δ0/ura3Δ0; leu2Δ0/leu2Δ0; his3Δ1/his3Δ1; met15Δ0/MET15; LYS2/lys2Δ0; YOL125w/YOL125w (TRM13)::kanMX4*. Y07126+OsTRM13: Y07126 strain transformed with *OsTRM13* cloned in pAUR101 chromosomal integrating shuttle vector (Takara Biotech). (B) PCR verification of positive yeast clones (OsTRM13-6, -11, -29) using *OsTRM13* gene specific primers. The PCR fragment size is 2281 bp. P C: positive control, NPB rice cDNA. (C) Relative levels of Am and Cm nucleosides in various yeast strains. Y07126 and Y27126 serve as negative and positive controls, respectively. The difference in Am or Cm nucleosides in Y07126 compared with Y27126 is shown in percentage. (D) Extracted LC-MS chromatograms of Am nucleosides in various yeast strains. See (C) for explanations. Error bars represent standard deviation from three technical replicates. *P<0.05 and **P<0.01 by Student’s *t*-test.

To corroborate the *in vitro* activity of OsTrm13, we expressed the *OsTRM13* gene in yeast strains deficient in Am nucleoside formation. Y07126 and Y27126 represent *Δtrm13* mutant and congenic wild-type, respectively, and defects of Am nucleoside were verified in Y07126 (Fig. 5). A full length *OsTRM13* cDNA clone in yeast chromosomal integrating vector was introduced into Y07126 to test whether *OsTRM13* could restore Am nucleoside. Three positive yeast clones were tested (OsTRM13-6, -11, -29) for the presence of the *OsTRM13* gene (Fig. 5B). tRNA was extracted subsequently from these lines, together with tRNAs from Y07126 and Y27126, and analysed for nucleoside modifications (Fig. 5C). The strain Y07126 carrying a knock-out allele of *trm13* had 15% Am nucleosides as compared with that of Y27126; however, the Y07126 clones that expressed *OsTRM13* had Am nucleoside levels similar to that of Y27126 (Fig. 5C). Indeed, in two of the clones (*OsTRM13-6*, *-11*) the Am nucleoside levels were even higher than that of Y27126. In addition, Y07126 also had reduced levels of Cm nucleosides, though not as substantial as that of Am nucleosides (since the Cm nucleosides are also present at other positions). Here, the Cm nucleoside levels were about 60% of that in Y27126 (Fig. 5D). Nevertheless, the Cm nucleoside levels were also fully restored in the *OsTRM13* expressing Y07126 clones (Fig. 5D). These data corroborate that OsTrm13 is an Am and/or Cm MTase.

### Increased expression of *OsTRM13* correlated with elevated Am nucleoside abundance during salt stress or ABA treatment

**Fig. 6.**
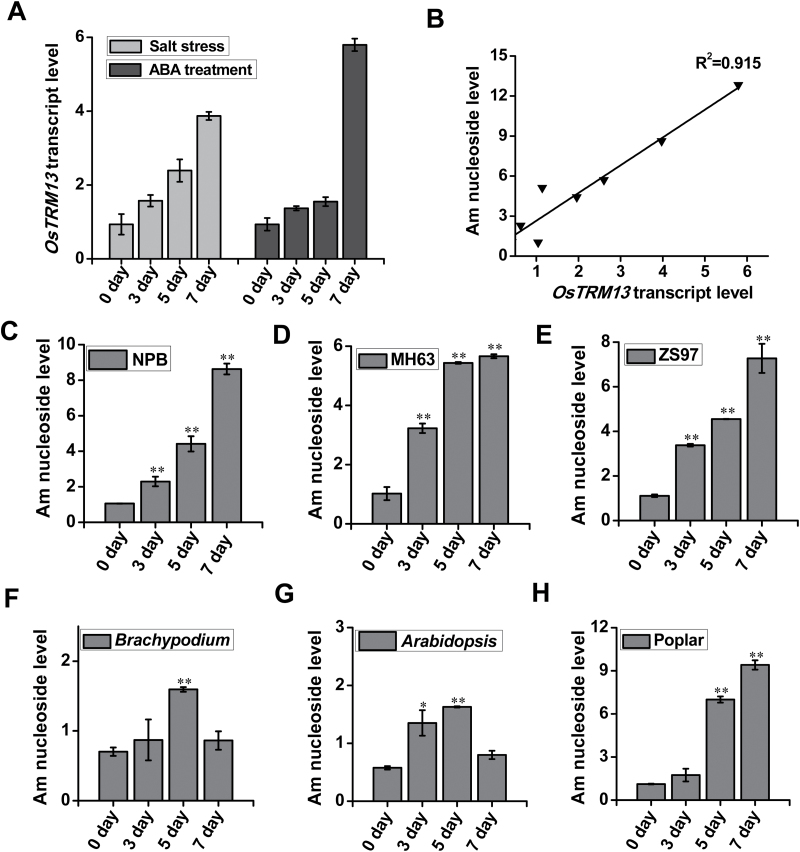
Salt stress and ABA treatment cause increase in *OsTRM13* expression and in tRNA nucleoside methylation. (A) *OsTRM13* transcript levels during salt stress (light gray) or ABA treatment (dark gray). (B) Fitting curve of Am nucleoside levels *vs OsTRM13* transcript levels under salt stress or ABA treatment. Pearson correlation coefficient (*R*^2^) was calculated with SPSS software. (C–H) Am nucleoside levels during salt stress in NPB (C), MH63 (D), ZS97 rice (E), *Brachypodium* (F), Arabidopsis (G) and hybrid poplar (H). For rice, *Brachypodium* and Arabidopsis, 10- to 14 day-old seedlings were used. For hybrid poplar, 1-month-old seedlings from tissue culture (*ca* 10 cm high) were used. Samples were taken at 0, 3, 5 and 7 d during salt stress treatment. tRNAs were extracted and nucleoside modifications were analysed. Error bars represent standard deviation from three biological replicates. *P<0.05 and **P<0.01 by Student’s *t*-test.

To evaluate whether the *OsTRM13* was induced by stress, we measured transcript levels during salt stress and ABA treatment. In addition, to see if the *OsTRM13* expression correlated with Am nucleoside levels, we also measured these levels under the same conditions. *OsTRM13* transcript level increased significantly during salt stress, as well as after ABA treatment (Fig. 6A). When we performed correlation analysis between transcript levels and Am nucleoside abundance, we observed a good correlation (Fig. 6B, *R*^2^=0.915).

To test if the increase of Am nucleoside was specific to Nipponbare rice accession, or if we could also observe such changes in other rice accessions, two other cultivated rice species, MH63 and ZS97, as well as *Brachypodium distachyon* as another monocot plant, were subjected to salt stress and analysed (Fig. 6C–H). For comparison, Arabidopsis and hybrid poplar were also tested. The results indicated that Am nucleoside increased during salt stress in all three rice accessions, as well as hybrid poplar (Fig. 6C–H). However, changes of Am nucleoside were not as pronounced in *Brachypodium* and Arabidopsis (Fig. 6F, G).

### OsTrm13 affects the endogenous Am nucleoside levels and impacts plant growth

**Fig. 7.**
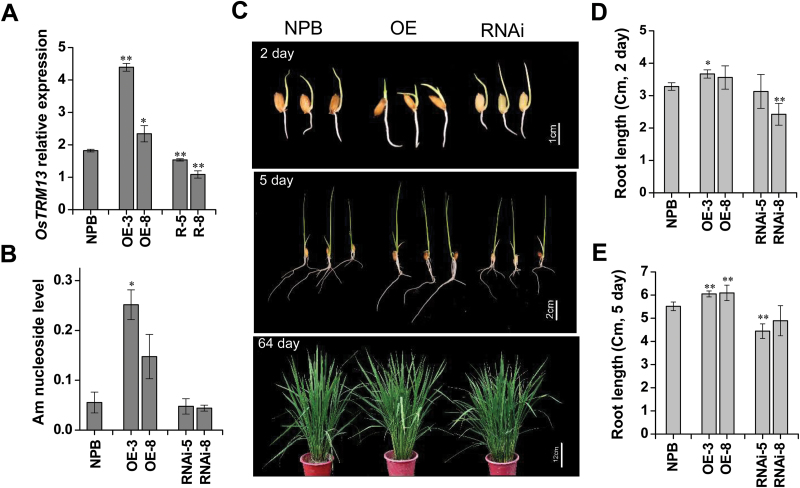
*OsTRM13* expression levels influence tRNA nucleoside modifications and rice growth and development. (A) Quantification of *OsTRM13* transcript levels in *OsTRM13* overexpression (OE-3, OE-8) and RNAi (RNAi-5, RNAi-8) transgenic lines. qRT-PCR was used to calculate gene relative expression, with *ACTIN* as reference gene. (B) Am nucleoside level in transgenic lines (OE and RNAi lines) compared with NPB rice. Total tRNA was extracted from 2-week-old rice seedlings and digested into nucleosides for LC-MS analysis. The Am nucleoside levels were calculated based on ion counts as percentage of the sum of the four canonical nucleosides, U, C, G and A. (C) Representative photographs of 2-day-old seedlings (upper panel), 5-day-old seedlings (middle panel) and mature plants (lower panel) of NPB, OE and RNAi transgenic plants. Scale bars are indicated in each photograph. (D) Quantification of root length of 2-day-old and 5-day-old rice seedlings of NPB, OE and RNAi transgenic plants. Ten seedlings were measured for each line. Error bars represent standard deviation from three biological replicates. *P<0.05 and **P<0.01 by Student’s *t*-test.

To investigate the impact of OsTrm13 on rice growth and development, we generated *OsTRM13* overexpression (OE) or RNAi transgenic rice. Since the endogenous *OsTRM13* expression is low in rice seedlings, RNAi plants only showed transcript reduction up to 40% (Fig. 7A). When tRNA nucleoside modifications were analysed, we found that the Am nucleoside levels were two to three times higher in the OE lines than that of control plants; however, the levels were not significantly different in the RNAi lines (Fig. 7B). This may be due to the fact that the Am nucleosides are not solely present on tRNAs, but also on snRNA, snoRNA and 5s rRNAs (Modomics and RNAmod databases). When seedling growth and root growth of the OE and RNAi lines were compared against wild-type, a slight increase in root length in 2- to 5-day-old *OsTRM13* OE seedlings was observed (Fig. 7C–E). Meanwhile, a significant decrease of root length was observed in the RNAi plants (Fig. 7C–E). Therefore, although a decrease of Am nucleoside in tRNAs from *OsTRM13* RNAi lines might be ‘masked’ by its presence on other small RNAs, the reduced root length in RNAi-8 lines (Fig. 7C, D) indirectly suggested that the tRNA-derived Am nucleosides was reduced. However, no differences in plant height were observed in later developmental stages, suggesting that the difference of Am nucleoside mainly affected early vegetative growth in rice.

### OsTrm13 is important for rice stress tolerance

**Fig. 8.**
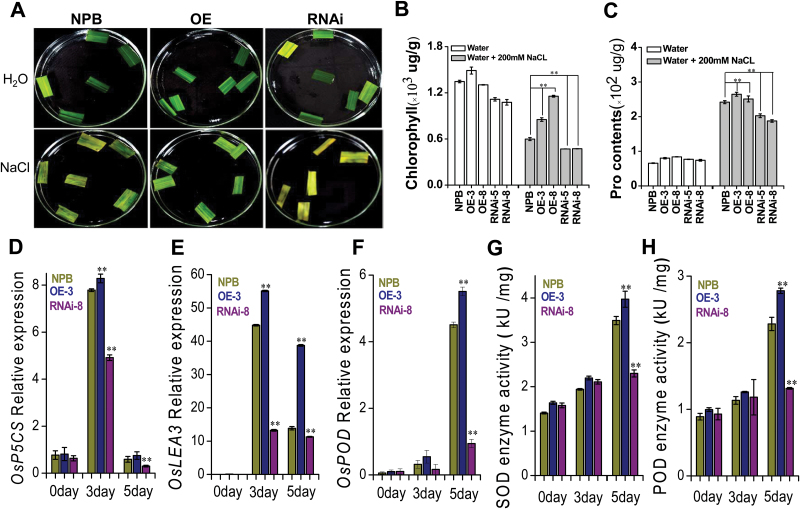
*OsTRM13* expression levels affect salt stress tolerance. (A). Photographs of flag leaves from NPB, *OsTRM13* OE-3 and RNAi-8 lines, before (H_2_O) or 3 d after salt stress treatment (200 mM NaCl). (B) Chlorophyll content of *OsTRM13* OE-3 and RNAi-8 transgenic lines before and after salt stress treatment. (C) Proline content measurements before and after salt stress. (D–F) Transcript levels of *OsPC5S*, *OsLEA3* and *OsPOD5* before and after salt stress. (G, H) SOD and POD enzymatic activities before and after salt stress. Error bars represent standard deviation from three biological replicates. *P<0.05 and **P<0.01 by Student’s *t*-test.

To assess how the changed levels of OsTrm13 and of Am nucleosides affected plant stress tolerance, we investigated the impact of salt stress on *OsTRM13* OE and RNAi transgenic plants (Fig. 8). Chlorophyll content was measured from flag leaves before or after salt stress (Srivastava *et al.*, 2016). As shown in Fig. 8A, B, NPB rice suffered *ca* 55% decrease of chlorophyll in response to 200 mM NaCl as compared with H_2_O treatment; however, the decrease of chlorophyll was much smaller in the OE lines, i.e. 33% and 11% in OE-3 and OE-8 lines, respectively (Fig. 8A, B). Moreover, the chlorophyll decrease in RNAi lines was more severe than the wild-type NPB rice (Fig. 8B).

Proline is an important osmolyte protectant for cells to mitigate osmotic stress, including drought and salt stress. *P5CS* is a key proline biosynthetic gene (You *et al.*, 2012) and its expression is, together with those of *LEA* and *POD* genes, an indicator of cell stress (Hasegawa *et al.*, 2000; Zhu, 2002; Duan and Cai, 2012; Nounjan *et al.*, 2012). Similarly, elevated enzymatic superoxide dismutase (SOD), peroxidase (POD) and catalase (CAT) activities were also used as markers for cellular stress (Hasegawa *et al.*, 2000). These parameters, i.e. proline content, transcript level of *OsP5CS*, *OsLEA3*, and *OsPOD5*, and SOD and POD enzymatic activities, were investigated in *OsTRM13* OE and RNAi transgenic plants that had been exposed to salt stress (Fig. 8C–H). We found that the proline levels in the OE plants were higher than in wild-type NPB, and that the *OsP5CS* transcript levels were higher, after 3 d of salt stress (Fig. 8C, D). *OsLEA3* expression also increased shortly after salt stress (Fig. 8E). In contrast, the expression of *OsPOD5* was induced later (Fig. 8F), suggesting a different regulatory window of the *POD*, *LEA*, and *P5CS* genes. Oxidative enzymes, such as SOD and POD, showed increased activity during the salt stress experiment, and the enzyme activities were generally higher in the OE lines than wild-type NPB, and lower in RNAi lines (Fig. 8G, H).

**Fig. 9.**
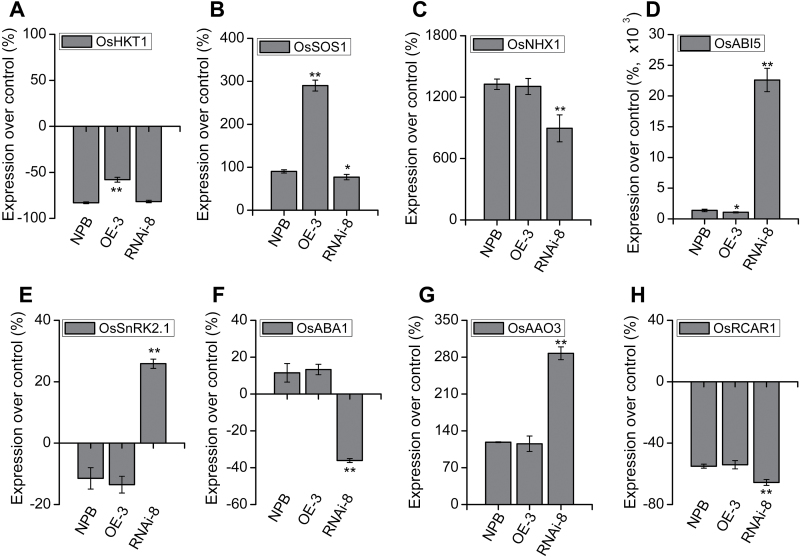
Expression analysis of ABA- and salt stress-related genes by real-time RT-PCR of NPB and *OsTRM13* transgenic seedlings after 3 d of salt stress. Relative expression of each gene was calculated using *ACTIN* as reference gene. The difference of expression level under salt stress condition *vs* control is indicated as percentage relative expression over control. (A, B, C) Expression over control (%) for transcript levels of *OsHKT1*, *OsSOS1* and *OsNHX1*. (D, E) Expression over control (%) for transcript levels of *OsABI5* and *OsSnRK2.1*. (F–H) Expression over control (%) for transcript levels of *OsABA1*, *OsAAO3* and *OsRCAR1*. Two technical replicates were run for each qRT-PCR reaction. Error bars represent standard deviation from three biological replicates. *P<0.05 and **P<0.01 by Student’s *t*-test.

ABA plays an essential role in abiotic stress, especially drought and salt stress (Fujita *et al.*, 2011; Nakashima and Yamaguchi-Shinozaki, 2013). We selected some of the salt stress marker genes (*SOS1*, *HKT1*, and *NHX1*) (Yokoi *et al.*, 2002; Hamamoto *et al.*, 2015), as well as genes involved in ABA synthesis, perception, and signaling (*ABA1*, *AAO3*, *PYL*/*PYR*/*RCAR1*, *ABI5*, and *SnRK2.1*) (Finkelstein and Lynch, 2000; Seo *et al.*, 2000; Xiong *et al.*, 2002; Yoshida *et al.*, 2002; Melcher *et al.*, 2009; Miyazono *et al.*, 2009; Santiago *et al.*, 2009), and measured their transcript levels. As shown in Fig. 9A, the transcript of *OsHKT1* (K^+^/Na^+^ influx) was down-regulated by salt stress in all three genetic backgrounds, but to a lesser extent in the *OsTRM13* OE-3 line (Fig. 9A). To the contrary, the transcript of *OsSOS1* (for Na^+^ efflux) was up-regulated in all three genetic backgrounds, and the most in the *OsTRM13* OE-3 line (Fig. 9B). Similarly, mRNA levels for *OsNHX1*, coding for a tonoplast-located Na^+^/K^+^ exchanger, were also increased (Fig. 9C). Here, the induction of *OsNHX1* transcript in the *OsTRM13* OE-3 line was comparable with NPB, but less in RNAi-8 lines (Fig. 9C). In contrast, the ABA signaling genes, *OsABI5* (bZIP TF) and *OsSASPK2* (SnRK2.1), were up-regulated in *OsTRM13* RNAi-8 lines, but less or slightly repressed in NPB and OE-3 lines (Fig. 9D, E). As for the ABA synthesis genes, the transcript level of *OsABA1* was slightly up-regulated in *OsTRM13* OE-3 lines as in the control, but down-regulated in RNAi-8 lines (Fig. 9F). *OsAAO3* transcript was up-regulated in all three backgrounds but the most in RNAi-8 lines (Fig. 9G), and *OsRCAR1* (ABA receptor) transcript levels were down-regulated in all, but also the most repressed in *OsTRM13* RNAi-8 lines (Fig. 9H).

Taken together, the expression data of ABA- and salt stress-related genes revealed a complex picture, but a partial explanation for how the *OsTRM13* OE-3 or RNAi-8 lines differed in salt stress tolerance. These differences appear to, at least in part, be due to variations in the expression of ion transporters (*HKT1*, *NHX1*, and *SOS1*), as well as genes in the ABA signaling pathway (*ABA1*, *AAO3*, *RCAR1*, *ABI5*, and *SnRK2.1*).

## Discussion

Rice is the staple food for a large fraction of the world’s population, and is thus an important cereal crop and a monocot model plant (Havukkala, 1996; Coudert *et al.*, 2010; Lo *et al.*, 2016). A basic understanding of how stress tolerance may be improved in rice is therefore of the utmost importance. We report that salt and ABA treatments induced Am nucleoside levels, and that *OsTRM13* is involved in Am nucleoside formation in rice. Furthermore, we show that changes in the *OsTRM13* expression levels contribute to salt stress tolerance of rice.

We found that OsTrm13 is an AdoMet-dependent MTase, which can catalyse Am and Cm modification at position 4 of yeast tRNA-Gly-GCC. The yeast Trm13p can use tRNA-Gly-GCC, tRNA-His-GUG, and tRNA-Pro-UGG as substrates (Wilkinson *et al.*, 2007). To find out the plant substrate tRNAs, we checked the tRNA genomic sequences from the Modomics and PlantRNA databases for yeast, Arabidopsis and rice. We found 40 tRNA-Gly, tRNA-His, and tRNA-Pro sequences in these species (Table 1). Rice contained 43 tRNA-Gly genes, coding for 18 unique tRNA-Gly sequences. Six of the tRNA-Gly-GCC sequences contained a cytidine at position 4, and one chloroplast gene had adenosine, making it a possible substrate candidate (Table 1). The three rice tRNA-His-GUG sequences all contained guanosine at position 4, and finally, all rice tRNA-Pro-UGG genes have either a cytidine or a guanosine at position 4 (Table 1). These data suggest that tRNA-Gly-GCC and possibly tRNA-Pro-UGG may be *in vivo* substrates for the OsTrm13. However, it is of course also possible that OsTrm13 may have extended its substrate repertoire and that also other tRNA nucleosides may be affected. Indeed, the OsTrm13 protein had a shorter MTase domain compared with Trm13p and AtTrm13, perhaps indicating a change in substrate recognition and activity.

Another major finding in this story is that *OsTRM13* transgenic rice influences salt stress tolerance. We analysed chlorophyll content, proline and MDA contents before and after salt stress in NPB control and *OsTRM13* OE or RNAi lines. We also measured enzymatic activities of oxidative enzymes, and relative expression of ABA and salt stress marker genes. The qRT-PCR analysis of ABA (*ABA1*, *AAO3*, *RCAR1*, *ABI5*, and *SnRK2.1*) and salt stress marker genes (*HKT1*, *NHX1*, and *SOS1*) gave a partial explanation for why *OsTRM13* OE or RNAi lines differed in salt stress tolerance (Figs 8 and 9). SOS1, as a Na^+^ efflux transporter, has a central role in salt stress tolerance, and the higher induction of *OsSOS1* in the *OsTRM13* OE-3 line (Fig. 9B) therefore supports an improved salt stress tolerance (Fig. 8A, B). These data are further supported by a lower reduction of *OsNHX1* in the *OsTRM13* RNAi-8 line (Fig 9C), which may reduce the buffering capacity of excess Na^+^ ions in the vacuole, leaving more Na^+^ in the cytosol and consequently a reduced salt tolerance phenotype (Fig. 8A, B). We also found differences in the expression of assorted ABA-related genes, e.g. *SnRK2.1*, *ABA1*, *ABI5*, *AAO3*, and *RCAR1*, in the OsTRM13 transgenic lines as compared with wild-type (Fig. 9D–H). Taken together, these data suggest that *OsTRM13* impacts the transcriptional regulation of ABA- and salt stress-related genes. Notably, our samples for gene expressions were taken 3–5 d after the salt stress treatment (Figs 8 and 9). Hence, our data are more likely related to the acclimation of the different lines to the salt stress response.

Stress is known to induce changes of tRNA nucleoside modifications in both prokaryotes and eukaryotes (Chan *et al.*, 2010). Here, we showed that Am nucleoside in tRNAs increased during salt stress in three rice accessions and poplar, but less in *Brachypodium* and Arabidopsis (Fig 6). A study by Kim *et al.* (2007) reported an increase of methylation index with salt stress. They found that the ratio of AdoMet and *S*-adenylyl-l-homocysteine increased during the early phase (first 12 h) of salt stress, accompanied by an induction of the biosynthesis of aromatic amino acids and lignin (Kim *et al.*, 2007). However, during long-term exposure to salt stress, methylation-related metabolites were repressed (Kim *et al.*, 2007). Since AdoMet is a methyl donor for Trm13p-like MTases, an increase of methylation during the early phase of salt stress might be associated with elevated levels of Am nucleosides presented in this study.

Our knowledge of how tRNA nucleoside modifications contribute to stress tolerance in plants is still largely lacking. We showed that both Am nucleoside levels and Am and/or Cm nucleoside MTase *OsTRM13* were up-regulated during salt stress or ABA treatment. Moreover, up- or down-regulation of *OsTRM13* influences the expression of ABA- and salt stress-related genes and therefore salt tolerance in rice. However, the primary effect of modified nucleosides on tRNA and translation might be translational instead of transcriptional. tRNA nucleoside modification could influence protein translation in three ways: (i) the location of the modified nucleoside on the tRNA molecule—in contrast to nucleosides within the anticodon loop that directly affect codon–anticodon recognition, Am on position 4 is more likely to influence the stability of the tRNA; (ii) the abundance of the tRNAs carrying the modification; and (iii) the codon composition of the target protein sequences. Proteomics data would be helpful to illustrate which proteins are affected by the presence or absence of Am nucleosides in *OsTRM13* transgenic plants during salt stress conditions. While we show that an increase of Am nucleoside during salt stress seems widespread in land plants, and that the protein sequences for the corresponding methyltransferase (Trm13p orthologs) are conserved (see Supplementary Fig S1), it is unclear whether the translational/transcriptional regulation behind the methylated nucleosides of tRNAs in ABA signaling- and salt stress-related proteins is similar in monocot and dicot plants.

## Supplementary data

Supplementary data are available at *JXB* online.

Fig. S1. Multi-sequence alignment of yeast Trm13p and plant TRM13 orthologs.

Table S1. LC-MS parameters for nucleoside analysis.

Table S2. Primers used in this study.

Table S3. Statistics of data for qRT-PCR and nucleoside abundance analysis.

## Authour contributions

YW performed most of the experiments; DL provided technical assistance for LC-MS analysis; XL conceived all bioinformatics analyses; JG, XJ and RZ performed vector construction, qRT-PCR and enzymatic activity experiments; ZH conceived transgenic plant analysis; BZ analysed the data; PC conceived the project and wrote the article with contributions of all the authors; SP supervised and complemented the writing.

## Supplementary Material

Supplementary_Figure_S1

Supplementary_Table_S1

Supplementary_Table_S2

Supplementary_Table_S3

## Abbreviations:

Ψ: pseudouridine
A: adenosine
ac^4^C: *N* ^4^-acetylcytidine
AdoMet: *S*-adenosyl-methionine
Am: 2′-*O*-methyladenosine
C: cytidine
Cm: 2′-*O*-methylcytidine
D: dihydrouridine
G: guanosine
Gm: 2′-*O*-methylguanosine
I: inosine
m^1^A: 1-methyladenosine
m^2^A: 2-methyladenosine
m^6^A: *N* ^6^-methyladenosine
m^5^C: 5-methylcytidine
m^1^G: 1-methylguanosine
m^2^G: *N* ^2^-methylguanosine
m^2^_2_G: *N* ^2^,*N*^2^-dimethylguanosine
m^7^G: 7-methylguanosine
m^1^I: 1-methylinosine
m^6^t^6^A: *N* ^6^-methyl-*N*^6^-threonylcarbamoyladenosine
m^5^U: 5-methyluridine
ncm^5^U: 5-carbamoylmethyluridine
t^6^A: *N* ^6^-threonylcarbamoyladenosine
U: uridine
Um: 2′-*O*-methyluridine.
