# Supplementary material for: The 2′-O-methyladenosine nucleoside modification gene OsTRM13 positively regulates salt stress tolerance in rice
Source: J Exp Bot. 2017 Mar 28;68(7):1479–91. doi: 10.1093/jxb/erx061 (PMC5444449; doi:10.1093/jxb/erx061)
Supplement: Supplementary_Figure_S1 [file erx061_suppl_Supplementary_Figure_S1.pptx]

## Slide 1
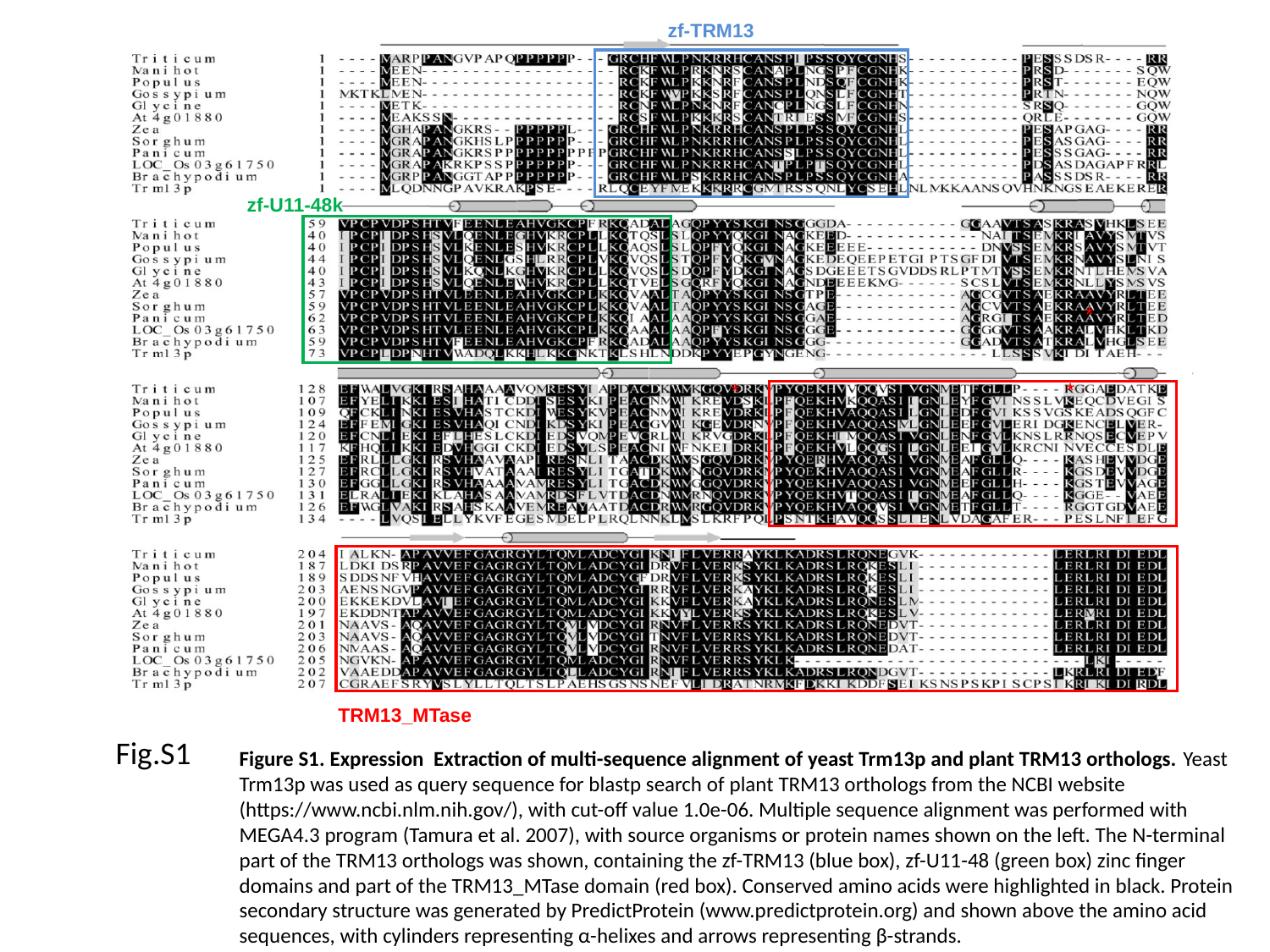

zf-TRM13
zf-U11-48k
*
*
*
TRM13_MTase
Fig.S1
Figure S1. Expression Extraction of multi-sequence alignment of yeast Trm13p and plant TRM13 orthologs. Yeast Trm13p was used as query sequence for blastp search of plant TRM13 orthologs from the NCBI website (https://www.ncbi.nlm.nih.gov/), with cut-off value 1.0e-06. Multiple sequence alignment was performed with MEGA4.3 program (Tamura et al. 2007), with source organisms or protein names shown on the left. The N-terminal part of the TRM13 orthologs was shown, containing the zf-TRM13 (blue box), zf-U11-48 (green box) zinc finger domains and part of the TRM13_MTase domain (red box). Conserved amino acids were highlighted in black. Protein secondary structure was generated by PredictProtein (www.predictprotein.org) and shown above the amino acid sequences, with cylinders representing α-helixes and arrows representing β-strands.
